# Social Media Use Among Young Adults With Connective Tissue Disorders: Cross-Sectional Pilot Study

**DOI:** 10.2196/16367

**Published:** 2020-10-30

**Authors:** Erin F Kelleher, Philip F Giampietro, Megan A Moreno

**Affiliations:** 1 Chicago Medical School Rosalind Franklin University of Medicine and Science North Chicago, IL United States; 2 Division of Medical Genetics Department of Pediatrics Rutgers Robert Wood Johnson School of Medicine New Brunswick, NJ United States; 3 Department of Pediatrics University of Wisconsin-Madison Madison, WI United States

**Keywords:** connective tissue disorders, social media, adolescents, young adults, Marfan syndrome, Ehlers-Danlos syndrome, Alport hereditary nephritis, Beals congenital contractual arachnodactyly, internet

## Abstract

**Background:**

Young people with genetic conditions often face challenges coping with their health condition. It can be difficult for them to meet someone with a similar condition, which is important for reinforcement of chronic illness management recommendations. Social media is used by 97% of young people in the United States and may provide those with these disorders a space for emotional expression and support. However, there is a scarcity of literature related to the use of social media among adolescents with genetic conditions as an indicator of their perception regarding their own condition.

**Objective:**

The purpose of this pilot study was to obtain preliminary data to assess and understand social media use by young people with connective tissue disorders and determine whether they use social media to connect with patients with similar conditions or whether they would be interested in doing so.

**Methods:**

We undertook a pilot study of selected connective tissue disorders occurring in young people between the ages of 11 and 25 years, including Marfan syndrome; Ehlers-Danlos syndrome subtypes classical, classical-like, cardiac-valvular, and vascular; Beals congenital contractual arachnodactyly; and Alport hereditary nephritis. The study took place within one pediatric clinical system. Patients were identified through electronic medical record search and International Classification of Diseases, Ninth Revision, coding at a Midwest university–based clinical system. Study subjects completed a short survey describing their experiences with their connective tissue disorders, their means of self-expression, their existing network of persons to communicate with, and their use of social media. Data analysis included nominal and bivariate regressions to compare social media use in relation to age.

**Results:**

Our 31 participants (42% response rate) were 55% female (17/31) and their average age was 18 years (SD 5). All participants used social media and there were no statistically significant differences between social media use and age. The majority of participants (25/30, 83%) reported that they never used social media to discuss their condition (*P*=.09), and only 17% (5/30) knew someone online with a similar condition (*P*=.50). Most participants (19/30, 63%) said they would communicate with someone with a similar disorder (*P*=.64).

**Conclusions:**

We found that young individuals with connective tissue disorders use at least one type of social media. A majority did not use social media to discuss their condition or know someone online with a similar condition. However, many persons were interested in finding others similarly affected. Social media could serve as a platform for young people with connective tissue disorders to connect. Peer support is important in disease management and adolescent development. Future studies should aim at understanding social media use among young people with connective tissue disorders and helping them connect with other people who have similar conditions.

## Introduction

Connective tissue disorders refer to a group of uncommon and heterogeneous conditions that are associated with pathogenic defects in the extracellular matrix [1-6]. As a consequence of these defects, affected individuals may have alterations in the development of bone, skin, vasculature, and other related organs [1-6]. A majority of connective tissue disorders are inherited in an autosomal dominant fashion [1-6].

Living with a genetic condition can lead to feelings of stigmatization and emotional pain due to being different from peers, in addition to social, emotional, and financial stress [7-9]. In particular, young adults with chronic illnesses often experience more social isolation compared to their peers [7]. Young people with Marfan syndrome have reported “difficulties in keeping up with peers” in school, sports, activities, relationships, and work because of their condition [9]. Time-consuming medical visits and treatments, fatigue, pain, and fear of injury were contributing factors to the difficulties associated with “keeping up with peers” [9]. Young people with Marfan syndrome also feel different from their peers due to their appearance, fatigue, pain, and disability. Many individuals with connective tissue disorders have to limit physical activities and sports to prevent life-threatening injuries [1-5]. Some adolescents and young adults experienced bullying, and individuals with lower self-esteem were more likely to avoid social activities, such as going to the beach or parties [9]. Previous research has found that having Marfan syndrome can lead to a lower quality of life mentally, due to emotional and psychological impairment [8-10].

Social media is used by 97% of US youth [11] and is recognized as an influential determinant of their health maintenance [12-14]. Social media can be used as an outlet to display people’s feelings and experiences regarding a health condition as well as foster social connection [14-16]. Additionally, social media makes it possible for teens to connect with new friends as well as maintain existing friendships [15,16]. The Pew Research Center found that 57% of teens between the ages of 13 and 17 years have made a new friend online [15].

Currently, social media sites provide support groups and/or posts for patients with connective tissue disorders included in this study. Posts and groups can be found by searching for the connective tissue disorder via the search bar on a social media site. There are groups and posts related to the more prevalent connective tissue disorders, such as Marfan syndrome and Ehlers-Danlos syndrome (Ehlers-Danlos), on Facebook, Twitter, Reddit, and Tumblr [17-20]. Less prevalent conditions, such as Alport hereditary nephritis (Alport syndrome) and Beals congenital contractual arachnodactyly (Beals syndrome), maintain smaller circles and have posts and groups on Facebook, Twitter, and Reddit [17-20].

Previous research has provided evidence that communicating about one’s condition can help individuals cope with their diagnosis, increase knowledge about their condition, and increase involvement in their own medical care [9,21-23]. Individuals with chronic illnesses who use social media to share and exchange information and experiences report an “enhanced feeling of self-worth and validation, often inhibited by living with chronic illness” [23]. For a majority, providing and receiving support through social media can decrease feelings of isolation and loneliness by bringing people together, especially when someone is not feeling well [23]. One individual with chronic pain explained, “social network sites have allowed me to have a social life...when the pain is bad, which is frequent, I cannot leave my house and spend time with friends” [23]. However, some individuals report that social media can have a negative impact due to feelings of withdrawal or frustration [23].

There is an increase in social media use among individuals with chronic illnesses to connect with others similar to them and learn information regarding their condition [12,13,24]. However**,** most young people with chronic conditions are particular about the content they publicly share on their social media accounts regarding their chronic condition [23,25,26]. Some adults with chronic illnesses feel a sense of control and an increase in self-worth when posting about their condition on social media, while others worry about the psychological and physical consequences of sharing personal information publicly [23].

Although a majority of young people receive positive feedback when posting about their condition, many chose not to post about their condition because they fear stigmatization and rejection [25,27]. Currently, there is a scarcity of literature regarding the number of young people with rare chronic illnesses that use social media to communicate about their condition.

Due to the rarity of connective tissue disorders, it can be challenging to meet other individuals with similar conditions. Social media allows users to find support globally by decreasing geographical and time barriers [23]. Some even use social media to connect individuals with health care providers around the world [23]. This is important for the treatment of individuals with rare conditions who do not have a specialist for their condition in their area. A prior study investigating Marfan syndrome social media references found that a majority of posts displayed personal experiences and symptoms regarding Marfan syndrome [28]. This study also found that Marfan syndrome was discussed across different public social media platforms. However, there is a scarcity of literature related to the use of social media among young people with genetic conditions as a window to their perception regarding their own condition. Based on this previous study, we hypothesized that young people with connective tissue disorders used social media to communicate their perceptions, experiences, and concerns regarding their condition.

The purpose of this pilot study was to obtain preliminary data to assess social media use, understand how social media was used, and determine whether patients with connective tissue disorders use social media to connect with other individuals with similar conditions or whether they would be willing to do so. With the understanding of how young people with selected connective tissue disorders communicate online, future research can be done on improving online support, since online communication keeps growing.

## Methods

### Overview

This cross-sectional pilot study was approved by the University of Wisconsin Health Sciences Institutional Review Board and took place from September 2013 to May 2015. The data were collected and analyzed at the University of Wisconsin-Madison, School of Medicine and Public Health, in the Department of Pediatrics, Division of Genetics and Metabolism.

A prior content analysis study investigating Marfan syndrome social media references found references to Marfan syndrome across public social media platforms, such as Instagram, Pinterest, Reddit, Tumblr, and Twitter [28]. However, there is a scarcity of literature regarding how young people with chronic genetic conditions, such as connective tissue disorders, use social media with regard to their condition.

### Participants

We identified young people between the ages of 11 and 25 years with connective tissue disorders, including Marfan syndrome, Ehlers-Danlos, Alport syndrome, and Beals syndrome; see Table 1 [1-6] for connective tissue disorder characteristics. Ehlers-Danlos subtypes include classical, classical-like, cardiac-valvular, and vascular Ehlers-Danlos. These connective tissue disorders were chosen due to their association with significant medical consequences.

**Table 1:** Summary of connective tissue disorders and associated features.

Condition (gene)	Worldwide prevalence	Non-life-threatening features	Life-threatening features	Key references
Marfan syndrome (FBN1)	1:5000-1:10,000	Tall stature Scoliosis Pectus excavatum or carinatum Lens dislocation Flat feet	Aortic root enlargement and dissection	[1]
Ehlers-Danlos syndrome^a^ (COL5A1, COL5A2, and COL3A1)	Classical: 1:20,000 Vascular: 1:50,000-1:200,000	Joint hypermobility Joint pain Soft velvety and/or elastic skin Easy bruising and poor wound healing	Aneurysms throughout the vascular tree (vascular subtype)	[2-4]
Alport hereditary nephritis (COL4A5)	1:50,000	Hearing loss Eye abnormalities (lenticonus) Edema in extremities	Loss of kidney function resulting in kidney failure	[6]
Beals congenital contractual arachnodactyly (FBN2)	Unknown	Contracted joints Scoliosis Crumpling of ear Pectus excavatum or carinatum Long, tall stature Mild aortic root enlargement	Aortic dissection (rarely seen) Rare, lethal cases can result in severe cardiovascular and gastrointestinal symptoms	[5]

^a^Combination of the four types: classical, classical-like, cardiac-valvular, and vascular Ehlers-Danlos syndrome; the prevalence for classical-like and cardiac-valvular Ehlers-Danlos syndrome is unknown.

**Figure 1:**
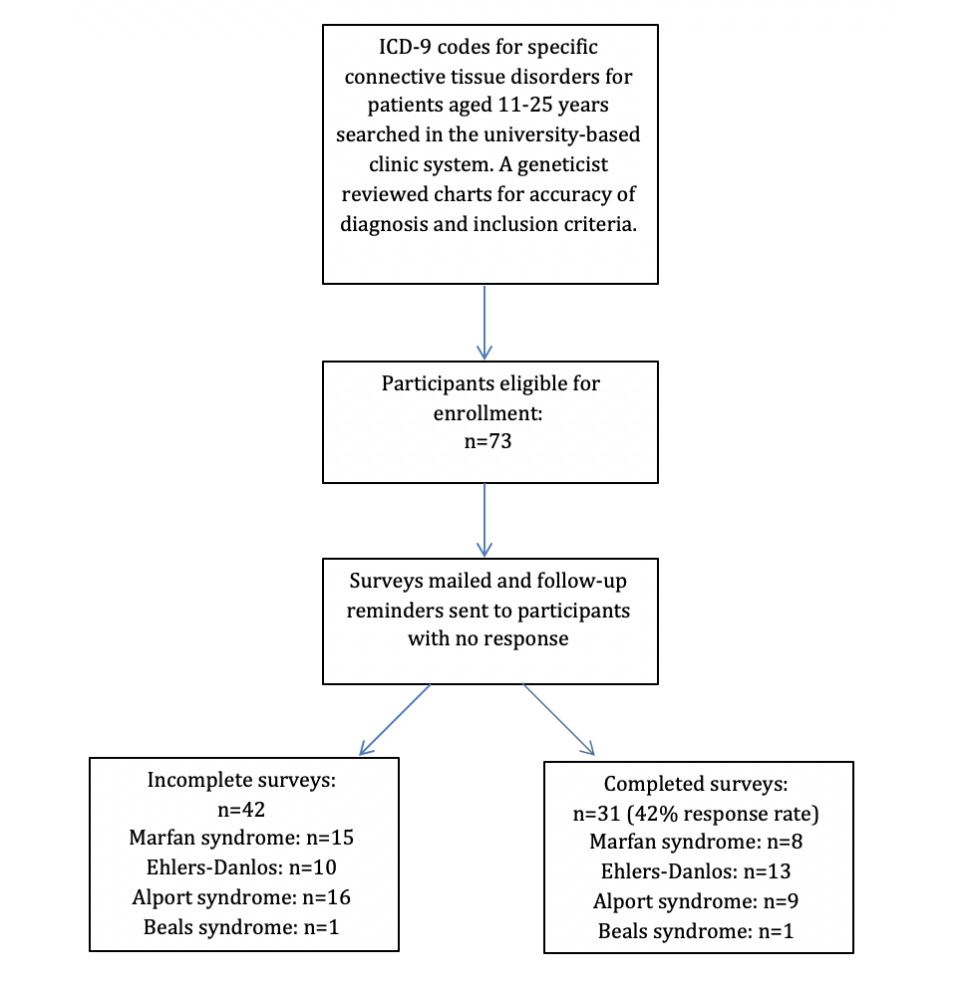
Study methods flowchart. Alport syndrome: Alport hereditary nephritis; Beals syndrome: Beals congenital contractual arachnodactyly; Ehlers-Danlos: Ehlers-Danlos syndrome; ICD-9: International Classification of Diseases, Ninth Revision.

Participants were queried in the University of Wisconsin Hospital and Clinics electronic medical record to identify patients who were classified in the different connective tissue diagnosis categories; see Figure 1 for the methods flowchart. The following information from medical records was reviewed by a coauthor and clinical geneticist (PG) for accuracy of diagnosis and qualification for enrollment in the study: medical complications related to connective tissue disorders related to the eye, heart, body skeleton, vascular system, skin, and spine as well as DNA testing that validates the diagnosis of Marfan syndrome and related connective tissue disorders. Charts were reviewed for patients between the ages of 11 and 25 years with Marfan syndrome; Loeys-Dietz syndrome; Ehlers-Danlos subtypes classical, classical-like, cardiac-valvular, and vascular; MASS (mitral valve, myopia, aorta, skin, and skeletal features) phenotype; Stickler syndrome; Alport syndrome; Beals syndrome; and dystrophic epidermolysis bullosa, regardless of gender and ethnicity. The patients with Loeys-Dietz syndrome, MASS phenotype, Stickler syndrome, and dystrophic epidermolysis bullosa with International Classification of Diseases, Ninth Revision (ICD-9) diagnosis codes did not meet the diagnostic criteria for the condition and, therefore, were excluded from this study. After reviewing medical charts, 72 patients met the inclusion criteria. All patients that met the study requirements were approached for possible enrollment.

### Survey Instrument

We reviewed previous literature to identify validated questions to assess social media use and created a self-developed survey [29]. The categories were developed with an adolescent health physician, a clinical geneticist, and adaptations from a Pew Research Center study [29]. In 2010, the Pew Research Center asked adolescents and young adults about social networking sites, gaming, and creative websites. We separated photo and video sharing from the Pew Research Center’s creativity section. Instagram was created in 2010 and, therefore, was not included in the Pew Research Center report. We felt that it was an important platform to include. We also wanted to include music sharing in the survey. The survey was piloted to genetic counselors at the institution and feedback was provided. The survey categories included the following: demographics, personal life (eg, hobbies, self-expression, and goals), basic information about their connective tissue disorder (eg, age of diagnosis, surgery and medication, and concerns), and social media use. To assess social media use, we asked participants about the number of hours per week spent on specific types social media sites; see Multimedia Appendix 1 for the survey.

The categories of social media included, and were described as, social networking sites (eg, Facebook, Twitter, etc), online gaming (eg, World of Warcraft), music sharing (eg, Pandora), video sharing or streaming (eg, YouTube and Hulu), photo sharing (eg, Instagram), and creative sites (eg, Pinterest and Tumblr). To understand how social media was used, we asked an open-ended question about how participants used social media. To determine whether participants used social media to connect with others with similar disorders, we asked participants about the frequency with which they discussed their condition and whether they received support from posting about their condition. To determine whether participants were willing to connect, we asked whether and how they would like to communicate with someone similar to them online.

### Recruitment

A data security analyst reviewed the electronic medical record to identify patients with connective tissue disorders. A clinical geneticist then reviewed records of potentially eligible patients to validate their fulfillment of the clinical criterion of having one of the connective tissue disorders being studied. Each patient was assigned a study code. After surveys were complete, the document linking the name of the patient with the study number was destroyed. All clinical data were kept under lock and key, and only the principal investigator and research assistant had access to the study.

A letter inviting each patient to participate in the study was signed by one of the patient’s physicians who participated in care for his or her underlying connective tissue disorder. Along with the letter, a consent and assent document, a survey, and a stamped and addressed return envelope were then mailed to the patient and their family. Participants who did not respond received a follow-up phone call several weeks after the initial letter of invitation. A follow-up letter and phone call was sent to participants several weeks after the first phone call. Participants were subsequently reminded about the study and informed that they could opt to take the survey on the phone. If there was no response, two additional follow-up letters were mailed home after the phone call. If at that time there was no response, no further contact was attempted.

### Data Analysis

Survey responses were recorded in a Microsoft Excel 2007 spreadsheet as a secure file on a protected server. Surveys were analyzed to determine descriptive outcomes described above. Survey questions that were not answered were not included in the analysis. Due to the wide age range and small sample size, responses between participants of differing connective tissue disorders were grouped together and analyzed by age group. The following categories were created: early adolescents (11-14 years), middle to late adolescents (15-21 years), and young adults (22-25 years) [30]. Data were analyzed using SPSS Statistics for Windows, version 26.0 (IBM Corp) [31]. A nominal regression was conducted to assess the relationship between age and whether participants would like to communicate with someone with a similar condition. Separate bivariate logistic regressions were used to analyze the relationship between age and discussing questions and concerns on social media, age and knowing someone offline, and age and knowing someone online. A Pearson correlation was calculated to determine the strength of the relationship between age and discussion of questions or concerns on social media.

## Results

### Participants

Of the 73 surveys sent to eligible patients, 31 surveys were completed and returned (42% response rate). The average age of nonrespondents was 18.4 years (SD 4.0), with a median age of 19 years. Among respondents who completed surveys, 55% (17/31) were female and 90% (28/31) were Caucasian, with an average age of 18 years (SD 5); see Table 2 for demographic characteristics. Out of 31 participants, 2 (6%) were Mexican and 1 (3%) was Lebanese. Over a quarter of the sample (9/31, 29%) were early adolescents, 35% (11/31) were middle to late adolescents, and 35% (11/31) were young adults. Only 2 participants out of 31 (6%) completed the survey over the phone. Out of 31 participants, 1 (3%) did not answer questions regarding social media use and was not included in the social media analysis.

**Table 2:** Demographic characteristics of the participants.

Characteristic		Early adolescents (n=9)	Middle to late adolescents (n=11)	Young adults (n=11)	Total (N=31)
**Gender, n (%)**					
	Male	4 (44)	5 (45)	5 (45)	14 (45)
	Female	5 (56)	6 (55)	6 (55)	17 (55)
**Age (years)**					
	Mean (SD)	13 (1)	17 (2)	24 (1)	18 (5)
	Median	13	17	24	17
**Race, n (%)**					
	White	7 (78)	10 (91)	11 (100)	28 (90)
	Other	2 (22)	1 (9)	0 (0)	3 (10)

### Survey

All participants indicated using at least one type of social media and 97% (29/30) used more than one type of social media. The most common social media types used were social networking sites (24/30, 80%) and video sharing (25/30, 83%). Creative sites (6/30, 20%), online gaming (8/30, 27%), music sharing (15/30, 50%), and photo sharing (15/30, 50%) were less popular social media sites. Although participants were not asked to specify which specific sites they used, Facebook, Twitter, Instagram, and YouTube were mentioned by participants throughout the survey. The most common way participants used social media was to maintain relationships (19/30, 63%), learn new information (7/30, 23%), and watch entertaining videos and photos (7/30, 23%).

There were no statistically significant differences in social media use based on age—age and discussion of questions or concerns about condition on social media: *r*=.32, *P*=.09 (N=30); age and knowing someone offline: χ^2^_1_=1.1, *P*=.30 (N=30); age and knowing someone online: χ^2^_1_=0.5, *P*=.50 (N=30); and age and wanting to communicate with someone: χ^2^_1_=0.9, *P*=.64 (N=30). Although there was statistical significance between age and frequency of posting online, there was a medium to moderate correlation. With a larger sample size, we hypothesize there could be a statistical significance in the frequency of posting about one’s condition at different ages.

Over one-third (11/30, 37%) of participants communicated their condition with a family member, 23% (7/30) with a health care professional, and 13% (4/30) with anyone (see Table 3). Only 17% (5/30) of participants discussed their questions and concerns on social media and 17% (5/30) used social media to communicate with someone with a similar condition. Only middle to late adolescents and young adults discussed concerns or communicated about their condition online. Of note, 3 of the 5 participants (60%) that discussed their condition on social media also used social media to communicate with someone with a similar condition. Over half (16/30, 53%) of participants knew someone with the same or a similar condition offline and only 17% (5/30) knew someone online with a similar or the same condition. Only 2 participants out of 30 (7%) knew someone both online and offline with a similar condition. However, 63% (19/30) of the participants answered that they would communicate with someone with a similar condition if they had the opportunity. Of those participants, 84% (16/19) suggested email or social media as a potential means of communication. Other suggestions included talking and meeting in person (6/19, 32%).

Ehlers-Danlos participants were the only 5 participants out of 30 (17%) that posted about their condition online, and all of them received support at least some of the time (3/5, 60%) or a majority of the time (2/5, 40%). A majority (4/5, 80%) of these participants that posted about their condition also knew someone online and communicated through email and texting.

**Table 3:** Online and social media survey results.

Question and responses		Early adolescents^a^ (n=9), n (%)	Middle to late adolescents (n=11), n (%)	Young adults (n=11), n (%)	Total (N=30), n (%)
**Who do you feel most comfortable talking to with regard to your condition?**					
	Family	4 (44)	2 (18)	5 (45)	11 (37)
	Health care professional	1 (11)	4 (36)	2 (18)	7 (23)
	Friend or significant other	1 (11)	2 (18)	1 (9)	4 (13)
	Anyone	1 (11)	1 (9)	2 (18)	4 (13)
	No one	0 (0)	1 (9)	0 (0)	1 (3)
	Other	2 (22)	2 (18)	1 (9)	5 (17)
**Do you discuss your questions or concerns about your condition on social media?**					
	Never	8 (89)	9 (82)	8 (73)	25 (83)
	Rarely	0 (0)	1 (9)	0 (0)	1 (3)
	Sometimes	0 (0)	0 (0)	3 (27)	3 (10)
	Always	0 (0)	1 (9)	0 (0)	1 (3)
**Do you use social media to communicate with people with similar conditions as you?**					
	No	8 (89)	8 (73)	9 (82)	25 (83)
	Yes	0 (0)	3 (27)	2 (18)	5 (17)
**Do you know anyone else from offline with the same or similar conditions?**					
	No	3 (33)	6 (55)	7 (64)	16 (53)
	Yes	5 (56)	5 (45)	4 (36)	14 (47)
**Do you know anyone else from online websites with the same or similar conditions?**					
	No	7 (78)	9 (82)	9 (82)	25 (83)
	Yes	1 (11)	2 (18)	2 (18)	5 (17)
**If you could, would you communicate with someone with the same or similar conditions?**					
	No	2 (22)	2 (18)	3 (27)	7 (23)
	Maybe	1 (11)	1 (9)	2 (18)	4 (13)
	Yes	5 (56)	8 (73)	6 (55)	19 (63)

^a^One participant did not answer all questions.

## Discussion

### Principal Findings

This study examined social media use of young people with connective tissue disorders. We ascertained that all of the participants used social media, and a majority used more than one type of social media site. Participants used social media to connect with friends and family, to learn new information, and as a source of entertainment. These findings support previous studies regarding social media use and motivations in young people without chronic conditions [15,16,32].

Young, middle, and older adolescents and young adults used social media similarly. A majority of young people utilized offline relationships, such as family or health care providers, rather than social media to discuss their concerns or questions regarding their condition. Early adolescents did not use social media to discuss their condition or communicate with other individuals with similar conditions. This supports a previous study that found that healthy young people aged 12-14 years were less likely to post about their health on social media compared to older adolescents and young adults [32]. Middle to late adolescents and young adults with Ehlers-Danlos were the only participants who used social media to discuss their condition.

A majority of participants that posted about their condition also used social media to communicate with someone with a similar condition. One explanation could be that individuals who have friends with similar conditions online may feel more comfortable posting about their condition. If young people see peers posting about their condition online, it may encourage them to post about their own condition. Participants who chose to post about their condition described receiving positive feedback from their online interactions. Previous studies have also found that positive feedback is received among the majority of the few young adults with chronic illnesses that post about their condition [25,27].

Social media allows users to communicate intimately and immediately, which has been shown to be an important component in social support [27]. Many individuals with chronic illnesses prefer to seek social support from someone with a similar illness [25,27]. Social media sites offer unprecedented opportunities for this type of support [23,25,27]. In order to successfully intervene with young people with connective tissue disorders to improve social connections, it is important to learn about the different ways young people use social media in regard to their condition. It is possible that young people with connective tissue disorders do not want to publicly post about their condition, but would be more willing to communicate about their condition in an anonymous or private way via private chatting or anonymous usernames [23]. Many young people with chronic illnesses are protective of their health information and do not disclose their health to extended family members and peers that are not close friends [25]. Therefore, many might not be comfortable publicly posting about their condition online.

Almost half of participants did not know another individual with a similar condition in their offline or online lives. However, we found a majority of participants were interested in communicating with other individuals with connective tissue disorders via email or social media. Our findings were consistent with previous studies that found that young people with chronic illnesses use social media, but not to discuss their condition [25,26]. Yet, a majority were interested in discussing their condition online with peers with similar conditions [25,26]. Young people may be more willing to meet individuals through social media in a private setting without sharing their health information with their entire social media circle [23]. Although we did not specifically inquire, a follow-up to this study could be to identify what would be necessary for these young people to connect with others regarding their disorder via social media.

We found that a majority of young people did not post about their condition online. This may be because many young people are scared of facing rejection, pity, or isolation when discussing their chronic condition online [25]. Previous research has found that few young people received negative feedback when posting online [25]. Perceived stigma felt by individuals with chronic disease can affect their willingness to post about their condition online [25]. Young adults are selective in the information they share and many want to seem like “regular young adults” among peers [26,32].

Young adults without chronic conditions are also concerned about their privacy when posting about their health on social media [32]. However, unlike young people with chronic conditions, healthy individuals are more likely to post about their health on social media [32]. Mood was the most common health topic discussed, followed by wellness and acute medical conditions [32]. These topics were discussed with the intention of finding peers with similar conditions, seeking advice, and receiving support [32].

Individuals with Marfan syndrome and related connective tissue disorders were polled at the 2019 Marfan Foundation Annual Conference about their favorite way to keep in touch with the Marfan syndrome and related connective tissue disorders community, and the majority of patients prefer communication through Facebook (unpublished data, 2019). This seems to be inconsistent with the survey findings reported in this communication. We speculate that patients feel less motivated to discuss their specific cases but are willing to discuss concerns related to the Marfan community as a whole using social media.

Due to the variety of social media platforms, it seems possible for individuals to find the type of connection or support they want without exposing their condition publicly. Different platforms offer varying levels of anonymity and different types of content. Platforms such as Reddit, Tumblr, and Twitter offer greater anonymity compared to Facebook. One way individuals are able control how much information is shared on their social media profiles is through privacy settings. In one study regarding social media use in teens with chronic illnesses, teens reported that they regularly check their privacy settings and feel confident in their privacy settings [26]. Having a sense of control over what information is shared is important to many young people with chronic illnesses [26]. Young people that post about their condition online have emphasized that they feel as though they are in control of what is shared [26].

Previous research has demonstrated that building relationships and networking with other individuals with a similar disorder has psychological and emotional benefits for people with connective tissue disorders [9,21-23,26]. Many young people do not think social media is helpful in obtaining health information [32]. Therefore**,** young people may prefer social media platforms that allow users to develop relationships and communities rather than sites that provide educational content. However, further research is needed to understand what barriers teens face or what processes would be helpful to connect them to others via social media.

Several studies have highlighted the importance of support among individuals experiencing stigmatization due to chronic illnesses [7,9,25,33,34]. A previous study found that 1 in 4 (25%) internet users with a chronic illness have gone online to find someone with a similar condition [35]. Peer support can increase peer interaction and participation and also offer authentic empathy and validation that professionals may not be able to offer [7,9,23,34]. Due to the rarity of connective tissue disorders, meeting someone offline with a similar condition may be challenging [1-6]. Social media could be used to overcome this challenge and bring together individuals with connective tissue disorders.

Young people without chronic illnesses use social media to meet new people with similar interests and maintain friendships [16]. Individuals with connective tissue disorders can use social media to identify other individuals with similar experiences [28]. However, young people with chronic conditions are less likely to publicly post about their condition and experiences on social media, which could make it difficult to find peers with similar conditions [25,26]. Young people with connective tissue disorders may need help finding spaces where they can connect with peers while maintaining their privacy regarding their condition.

### Limitations

This study has several limitations. This pilot study was conducted from 2013 to 2015. The internet and social media are constantly changing and, therefore, social media patterns in this study may be different than current social media trends. This study was performed using a single hospital network. Our study was composed of a small sample size of individuals with a limited number of connective tissue disorders. A majority of the sample was Caucasian and, therefore, results cannot be generalized to all individuals with connective tissue disorders. We realize there is clinical genetic heterogeneity among these conditions, especially among the Ehlers-Danlos subtypes. Future studies should be aimed at surveying young people among the different Ehlers-Danlos subtypes. It is also possible that there was selection bias in this sample population. However, regardless of race, ethnicity, and socioeconomic status, a majority of young people are on social media already and are, therefore, familiar with using it [16]. Despite these limitations, social media relationships are becoming more common in young people and, therefore, could be used to form relationships for young people with connective tissue disorders [15].

### Future Studies

In this pilot study, we did not directly survey why participants may have chosen not to communicate online about their diagnosis or whether they passively use social media in regard to their condition. Future research can address possible reasons for this, such as lack of participation with health care professional–approved sites, lack of patient interest, or passive social media use, etc. Future studies may also address what would be necessary in order for patients to use social media as a vehicle to communicate individual perceptions regarding their condition. Further research efforts should focus on whether young people would be interested in private social media settings to meet and interact with peers. Future studies could also focus on comparing adults and children with genetic conditions and their social media use.

### Conclusions

Although there are social media spaces for individuals with connective tissue disorders to post on and follow, we found that a majority of young people in this study did not use social media to discuss or communicate with others about their genetic condition. However, a majority of participants were interested in communicating with individuals with similar conditions. Due to the rarity of connective tissue disorders, many young people do not know someone with a similar condition. For many individuals, having social support can be instrumental in coping with a chronic illness. Peer mentorship regarding a chronic disease not only aids in disease management and social isolation reduction, but is also a positive factor for adolescent development.

## Appendix

Multimedia Appendix 1 Survey mailed to participants.

## Abbreviations

Alport syndrome: Alport hereditary nephritis
Beals syndrome: Beals congenital contractual arachnodactyly
Ehlers-Danlos: Ehlers-Danlos syndrome
ICD-9: International Classification of Diseases, Ninth Revision
MASS: mitral valve, myopia, aorta, skin, and skeletal features
