# Supplementary material for: Social Media Use Among Young Adults With Connective Tissue Disorders: Cross-Sectional Pilot Study
Source: JMIR Pediatr Parent. 2020 Oct 30;3(2):e16367. doi: 10.2196/16367 (PMC7665944; doi:10.2196/16367)
Supplement: Multimedia Appendix 1 [file pediatrics_v3i2e16367_app1.docx]

Participant’s ID number ______________________

Date_____________________

**Demographics:**

1) Gender: 🞎 Male 🞎 Female

2) Age: Years: ____ Months: ____

1. Racial Categories: [*Check all that apply*]

🞎 American Indian/Alaska Native 🞎 Black or African American

🞎 Asian 🞎 White

🞎 Native Hawaiian or Other Pacific Islander 🞎 Other, *specify*:_______

1. Ethnic Category:

🞎 Hispanic or Latino 🞎 Not Hispanic or Latino

**Questions regarding your personal life:**

1. What activities do you enjoy participating in outside of school?  These could include clubs, sports, community or other activities.

________________________________________________________________________________________________________________________________________

1. In what ways do you express yourself related to your feelings?

________________________________________________________________________________________________________________________________________

1. In what ways do you exercise?

________________________________________________________________________________________________________________________________________

1. How often do you exercise?

Never  Rarely  Sometimes  Most of the time  Always

1. What are your goals for the future?

________________________________________________________________________________________________________________________________________

**Questions regarding Marfan syndrome^a^:**

1. How old were you when you were diagnosed with Marfan syndrome? _______________
2. Have you ever had surgery?  Yes  No
   1. If yes, please list ______________________________________________
3. Are you currently taking any medications?  Yes  No
   1. If yes, please list ______________________________________________
4. What are your biggest concerns relating to Marfan syndrome? ________________________________________________________________________
5. Who do you feel most comfortable talking to with regards to Marfan syndrome? ______________________________________________________________________

**Questions relating to social media use:**

1) How many hours do you spend per week on each social media website?

| Social Media Sites | Social Networking sites (Facebook, Twitter, etc) | Online gaming (WoW) | Music Sharing (Pandora) | Video sharing or streaming (YouTube, hulu) | Photosharing (Instagram) | Creative sites (pintrest, tumblr) |
| --- | --- | --- | --- | --- | --- | --- |
| Hours per week |  |  |  |  |  |  |

2) What are the benefits and disadvantages of social media websites?

| Benefits | Disadvantages |
| --- | --- |
|  |  |

3) In what ways do you use social media?

|  |
| --- |

1. Do you discuss your questions or concerns about your condition on social media?
   1. Never  Rarely  Sometimes  Most of the time  Always
2. Do you get support from others on social media when you post about your condition?
   1. Never  Rarely  Sometimes  Most of the time  Always
3. Do you use social media to communicate with people with similar conditions as you?   Yes  No
   1. If yes, please explain the types of relationships you have formed using social media ________________________________________________________________________________________________________________________
4. Do you know anyone else from offline with Marfan syndrome or other similar conditions?  Yes  No
5. 8) Do you know anyone else from online websites with Marfan syndrome or other similar conditions?  Yes  No
6. If yes, in what way(s) do you communicate?____________________________________________________________
7. If you could communicate with someone with Marfan syndrome, would you?

Yes  No

1. If yes, what would be your preferred way(s) to communicate?_____________________________________________________
2. Would you like to be able to communicate with your doctor online  Yes  No
3. If yes, what would be your preferred way(s) to communicate?_____________________________________________________
4. Are you willing to be contacted by the study team in the future about additional studies on Marfan syndrome and social media?  Yes  No

^a^Connective tissue disorder corresponded with connective tissue disorder of participant. Connective tissue disorders surveyed included Marfan syndrome, Ehlers-Danlos subtypes, Alport’s hereditary nephritis, and Beals congenital contractual arachnodactyly.
